# Bibliometric analysis of lupus nephritis in children from 1999 to 2022: A review

**DOI:** 10.1097/MD.0000000000036670

**Published:** 2024-01-05

**Authors:** Yunhong Ma, Shuangyi Wang, Fei Luo, Yuan Zhang, Juanjuan Diao

**Affiliations:** a College of Chinese Medicine, Shandong University of Chinese Medicine, Jinan, China; b Department of Pediatrics, Affiliated Hospital of Shandong University of Traditional Chinese Medicine, Jinan, China; c First College of Clinical Medicine, Shandong University of Traditional Chinese Medicine, Jinan, China.

**Keywords:** bibliometric analysis, children lupus nephritis, Citespace, hot spot, VOSviewer

## Abstract

Lupus nephritis (LN) is a complication of systemic lupus erythematosus and a damaging disease of the kidney. The injury of LN in children is more serious than that in adults. However, the literature in this field is numerous and complex, which brings great challenges for researchers to extract information. The purpose of this study is to carry out bibliometric analysis and visualization of published literatures, and identify current research hotspots and future research trends in this field. Literature was retrieved from the Web Of Science database from 1999 to 2022. The literature was analyzed and visualized using Citespace 6.1.R6, VOSviewer 1.6.18, and Microsoft Excel 2019. A total of 1059 articles were included in this study. In the past 13 years, an increase in the number of publications every year. Brunner HI is the author with the highest number of published and cited papers in this field, followed by Wenderfer SE. The United States and China are the countries with the highest number of published papers. University Toronto is the most productive institution, followed by University Cincinnati. The most prolific journal was Pediatric nephrology (IF 2.67), followed by lupus (IF 2.21). Lupus was cited the most, followed by Pediatric nephrology. The keyword burst showed the earliest and longest burst was antiphospholipid antibody, validation/risk/rituximab/safety is the current research hotspot. The article with the highest number of citations was Hochberg MC 1997 published in Arthritis Rheum. This study provides valuable information summary for the field of LN in children, which is helpful to strengthen the cooperation among countries, institutions and authors, and promote the research in the field of LN in children.

## 1. Introduction

Systemic lupus erythematosus (SLE) is an autoimmune disease affecting multiple organs and systems throughout the body. Its clinical manifestations are fever, fatigue, butterfly erythema, albuminuria, hematuria and kidney injury, etc. The clinical manifestations of systemic lupus erythematosus in children are similar to those in adults, but the early onset is accompanied by more severe organ damage.^[1,2]^ Lupus nephritis (LN) is a serious complication of systemic lupus erythematosus, which is also an important factor affecting the quality of life of affected children. Studies have shown that 60–80% of children with SLE will develop LN.^[3]^ In addition, several studies on renal outcomes have demonstrated that the severity of LN in childhood is higher than that in adults.^[4]^ In recent years, more and more studies have been conducted on LN in children. But compared with adults, kidney biopsy in children is still rare, and new biomarkers have not been allowed to be used in the treatment of LN,^[5]^ which leads to a bottleneck in the development of LN in children. Although the number of literatures is large, they lack of innovation. In addition, there is no summary article in this field at present. The purpose of this article is to make statistical analysis of the published literature in the field of LN in children, so as to provide help for the future diagnosis and treatment of LN in children.

Bibliometrics is an interdisciplinary science that integrates mathematics, statistics and documentation and quantitatively analyzes all knowledge carriers.^[6]^

## 2. Materials and methods

### 2.1. Data selection

The papers included in this study were sourced from the Web Of Science (WOS) database, which is known for its coverage of authoritative, high-impact academic journals worldwide. It provides comprehensive citation index records and primarily focuses on the natural sciences.^[7]^ Several studies have demonstrated that WOS offers higher accuracy and periodicity compared to databases like Scopus. While Science Direct encompasses a wide range of journals, it has a relatively smaller number and variety of journals compared to the WOS database. Although PubMed is the central search platform for biomedicine, it is typically a abstract-based database that lacks citation information. As a result, WOS is widely regarded as the most suitable database for bibliometric analysis.^[8]^ All the literature used in this study was retrieved and downloaded on November 17, 2022. The search strategy employed was TS = (“Lupus nephritis” OR “Lupus kidneys” OR “lupus nephropathy” OR “human lupus nephritis” OR “systemic lupus erythematosus nephritis” OR “lupus-glomerulonephritis”) AND TS = (Children OR Childhood OR Pediatric*). The search period spanned from 1999 to 2022, a total of 1123 articles were retrieved, and 1056 articles were included by selecting English literature and review articles. Two researchers (M and W) independently analyzed the data, which included title, abstract, keywords, author, institution, country, journal, references, and publication citations. In case of any differences of opinion, a third researcher (D) resolved them through independent negotiation and discussion. The complete records and references were output as plain text.

### 2.2. Statistical analysis

CtieSpace is a citation visualization software based on JAVA, which creates visual maps for research content and is mainly used to help literature visualization and analyze the knowledge base and frontier hot spots in the research field.^[9]^ Due to its rich functions, Citespace has become a popular method for medical big data analysis.^[10–13]^ This research is used for cluster analysis of co-cited literature and analysis of centrality between countries. The time span (1999–2022), years per slice (1), links (strength: cosine, scope: within slices), selection criteria (maximum number of selected items per slice = 50), pruning (Pathfinder, Pruning sliced networks), and all of the other parameters were left at their default settings. Node centrality is a quantification of the importance of a research point, which represents the number of connections of the node. A higher centrality indicates that there are more connections passing through this node in the network, which reflects that this node acts as an important bridge connection in this research network. Cluster analysis of co-cited references will identify highly influential references in this field. The modularity Q > 0.3 and mean silhouette > 0.5 indicate that the clustering results are sufficient and credible.

VOSviewer is a free analysis software that can carry out literature visualization. It was developed by van Eck and Waltman. Through cluster analysis, it can more intuitively display the cooperative network relationship among various analysis contents, and present large-scale bibliometric mapping in an easy to explain way.^[14]^ This study is used to deal with the cooperative network relationship between authors/institutions/journals/countries, and through the size of nodes, we can intuitively see the size difference of data, and we extract and cluster the occurrence frequency of keywords. Different colors represent different clustering networks. In this study, the parameters of the VOSviewer were as follows: The counting method was selected for “full counting.” The minimum number of citations for the co-cited authors and co-cited references was 20 and 30, respectively. The unit of analysis of the co-occurrence keyword was “all keyword,” and the threshold for the minimum number of occurrences was set to 100. The thresholds for the minimum number of occurrences of co-occurring institutions and co-occurring authors were set to 15 and 15.

In this study, Citespace 6.1.R6 and VOSviewer 1.6.18 are used to make statistical analysis of literature information from WOS core database and draw visualization map. Microsoft Excel software is used to summarize and map the annual number of published documents and the annual cumulative number of published documents.

## 3. Results

### 3.1. Publication output and temporal trend

**Figure 1.**
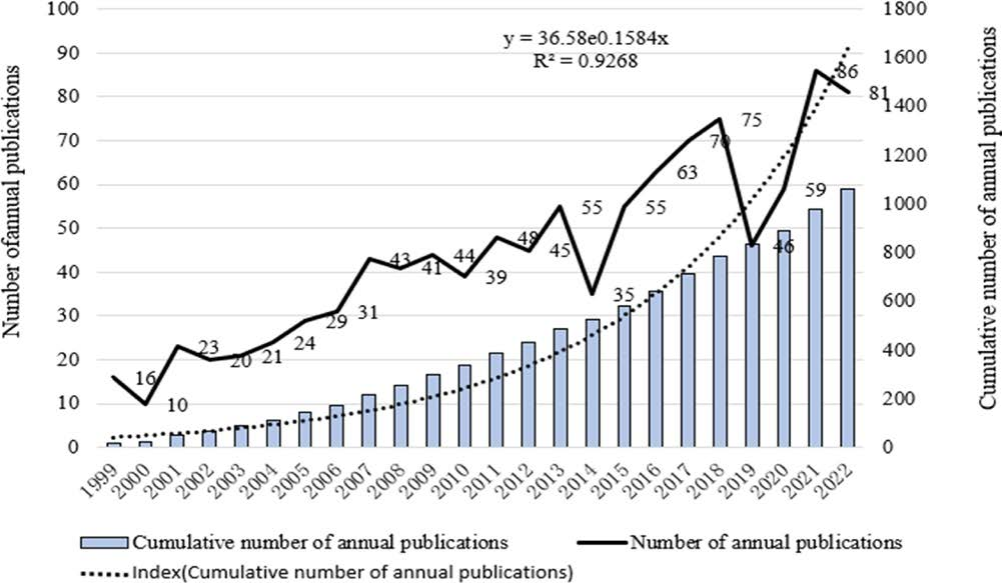
Trend of publications in the field of lupus nephritis in children (1999–2022).

From 1999 to 2022, Web Of Science included a total of 1059 literatures on childhood LN, including 884 articles and 175 reviews. Figure 1 describes the specific number and annual trend of publications on childhood LN from 1999 to 2022. Except for 2014 and 2019, Although the number of publications decreased slightly in 2014 and 2019, they still had higher research value compared with the previous publications. The number of publications increased rapidly from 2019 to 2021, which indicated that the number of publications reached a peak. In the past 2 years, researchers have paid close attention to LN in children, which is of great significance for the development of this field. In addition, an exponential function *y* = 36.58e0.1584*x (R*² = 0.9268, where *X* is the year and *Y* is the annual cumulative publication) was created to further evaluate the trend in the study of LN in children.

### 3.2. Analyze of authors and co-cited authors

**Figure 2.**
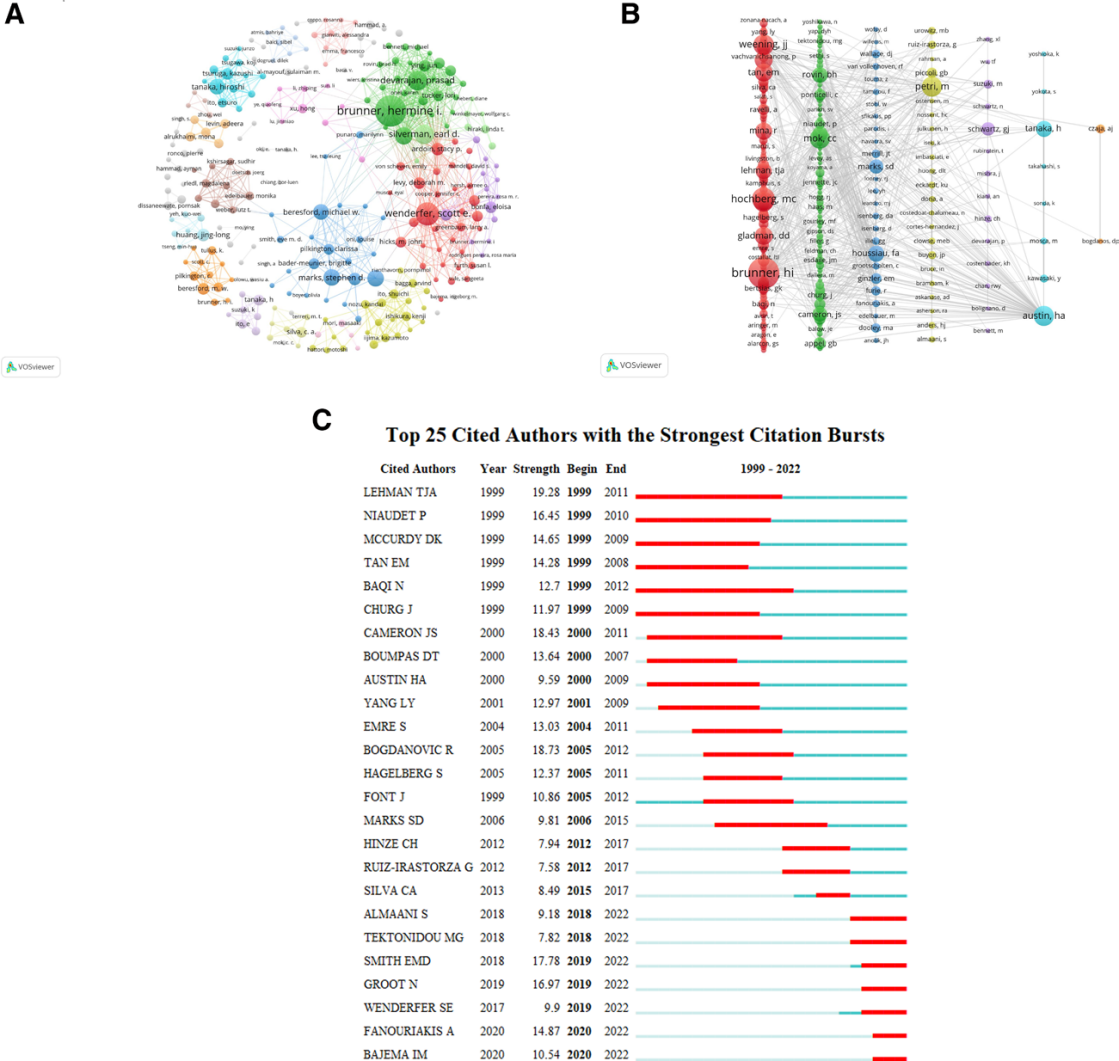
Analyze of authors and co-cited authors. (A) Visualization of authors. (B) Visualization of cited authors. (C) The top 25 cited authors with the strongest citation bursts.

From the perspective of author analysis, the ranking of top 10 prolific authors in the past 23 years is shown in Table 2. Brunner Hermine I of Cincinnati Children’s is the most prolific author, with 26 publications in total. It was followed by Wenderfer SE from Baylor College of Medicine, who published 21 articles in total. Devarajan Prasad, also from Cincinnati Children’s, ranked third, published 12 articles in total. Five of the top 10 authors belong to the US and 3 to the UK, which shows that the US and the UK have outstanding contributions in this field. The authors most frequently cited in the article references are the key indicators of author contribution available. Citations represent the number of times an author’s work has been cited, Table 1 shows the top 10 most cited authors in the references, all of whom have been cited more than 150 times. Brunner HI is the first cited author, followed by Hochberg MC, Weening JJ. Brunner HI ranked first in the number of published papers and cited times, which fully indicates that the authors have made outstanding contributions in this field. Brunner HI and her team worked on noninvasive clinical and laboratory measurements that accurately reflect LN activity and response to therapy, levels of neutrophil gelatinase-associated lipocalin (NGAL) were moderately associated with activity in LN. Urinary NGAL is a predictive biomarker of acute kidney injury, a substance produced by excessive induction and expression of renal tubular epithelium.^[15]^ Although Brunner·HI demonstrated that levels of NGAL are associated with activity in LN, due to research limitations, it is not certain whether urinary NGAL can predict early activity in LN.^[16,17]^ We expect this conclusion to be confirmed in future studies. In addition to urinary NGAL, Brunner HI demonstrated that urinary transforming growth faction-β and ceruloplasmin, biomarkers such as alpha-1-acidic glycoprotein, lipoprotein-like prostaglandin D synthase, transferrin or vitamin D-binding protein can predict the effectiveness of treatment for LN.^[18,19]^ Wenderfer SE found that children with active LN had higher urine CD163 levels than children without active LN and healthy children. Furthermore, urinary CD163 levels were significantly correlated with SLEDAI, renal SLEDAI, urinary protein excretion, and C3 complement levels. Meanwhile, urine CD163 is also associated with high renal pathological activity index and chronic index, and is closely associated with interstitial inflammation and interstitial fibrosis according to simultaneous renal biopsy.^[20]^ The author collaborative network visualization is performed by the VOSviewer, as shown in Figure 2A, where each color represents a collaborative network and the node size represents the magnitude of the frequency, Brunner HI; Wenderfer SE; Marks SD; Tanaka Hiroshi belong to different cooperative networks and occupy dominant positions. Inter-network cooperation also exists among Brunner HI, Wenderfer SE, Marks SD. A visualization of the cited authors is shown in Figure 2B, from which it can be seen that among the top 10 cited authors, Brunner HI; Petri M; Mok CC; Austin HA is dominant in their respective clusters. It can be seen from Figure 2C that among the strongest citation bursts cited by the top 25 authors from 1999 to 2022, Lehman TJA had the highest intensity and Baqi N had the longest duration.

**Table 1:** Top 10 authors of the number of publications.

Rank	Authors	Documents	Authors	Citations
1	Brunner, Hermine I.	32	Brunner, HI	425
2	Wenderfer, Scott E.	22	Hochberg, MC	255
3	Devarajan, Prasad	18	Weening, JJ	218
4	Silverman, Earl D	17	Hiraki, LT	209
5	Marks, Stephen D.	16	Petri, M	209
6	Klein-Gitelman, Marisa S.	16	Mok, CC	208
7	Tullus, Kjell	15	Austin, HA	204
8	Beresford, Michael W.	14	Tan, EM	170
9	Tanaka, Hiroshi	13	Tucker, LB	169
10	Ardoin, Stacy P.	12	Gladman, DD	169

**Table 2:** Top 10 countries of the number of publications.

Rank	Countries	Count	Countries	Centrality
1	United States	292	Netherlands	0.15
2	China	147	Germany	0.11
3	Japan	96	France	0.10
4	Canada	81	Canada	0.09
5	England	78	England	0.08
6	Italy–Brazil	60	Italy	0.08
7	Brazil	55	Scotland	0.08
8	France	51	United States	0.05
9	India	49	Belgium	0.05
10	Turkey	49	Saudi Arabia	0.04

The visualization analysis of countries and institutions is performed by Citespace and VOSviewer. As can be seen from Table 2, the United States has the largest number of publications with 292 articles, followed by China with 147 articles and Japan with 96 articles. Canada and the United Kingdom also have a high number of publications, indicating a high level of research interest in this field in these countries. As shown in Figure 3A, the centrality of the Netherlands, Germany, and France exceeds 0.1, and the nodes are shown as purple rings, suggesting that these 3 countries cooperate closely with other countries in the field of LN in children and act as a bridge in national communication. The different color clusters in Figure 3C represent different cooperation networks. In the green cluster, the United States dominates, with frequent cooperation with Canada, the United Kingdom, Italy, and Brazil, as well as close ties with Japan and India. In the blue cluster, China dominates. It has close cooperation with the United States, as well as with Britain, Canada, France, and Japan. The red cluster is dominated by England, which has frequent cooperation with Italy and the United States, close relations with France, Canada, and China, as well as cooperation with the Netherlands. The size of nodes in Figure 3B represents the size of frequency, and the larger the node is, the more the number of publications of institutions. Table 3 shows the top 10 institutions and the countries where they are located. Univ Toronto (42) leads in the number of publications, followed by Univ Cincinnati (35) and Univ Sao Paulo (33). Five of the top 10 publishing institutions are based in the United States, which shows that the country has a strong research power in this field. Figure 3D visualizes the cooperative network between institutions, Univ Toronto and Univ Cincinnati in the red cluster; Hosp Sick Children has a close relationship with Cincinnati Childrens Hosp Med CTRC, and has more cooperation with Ohio State Univ. Univ Liverpool and Cincinnati Childrens Hosp Med CTRC and Great Ormond St Hosp Sick Chil in the blue cluster and Alder Hey Childrens NHS Fdn Tr is closely related; in the green cluster, Mayo Clin has cooperated with Univ Toronto, Univ N Carolina, and Columbia Univ. In yellow cluster, Hirosaki Univ is frequently connected to Hirosaki Univ Hosp. Combined with Figure 3D and Table 3, we know that among the top 10 institutions in the publication ranking, 8 institutions are in the cooperative network of the red cluster, which indicates that the cooperative network of the red cluster has a high influence in the field of lupus kidney in children.

**Table 3:** Top 10 institutions by number of publications.

Rank	Institutions	Count	Original country
1	Univ Toronto	42	Canada
2	Univ Cincinnati	35	United States
3	Univ Sao Paulo	33	Brazilian
4	Cincinnati Childrens Hosp Med CTRC	28	United States
5	Hosp Sick Children	27	Canada
6	Univ Liverpool	26	England
7	Childrens Hosp Philadelphia	25	United States
8	Ohio State Univ	24	United States
9	Hirosaki Univ	23	Japan
10	Texas Childrens Hosp	23	United States

**Figurer 3.**
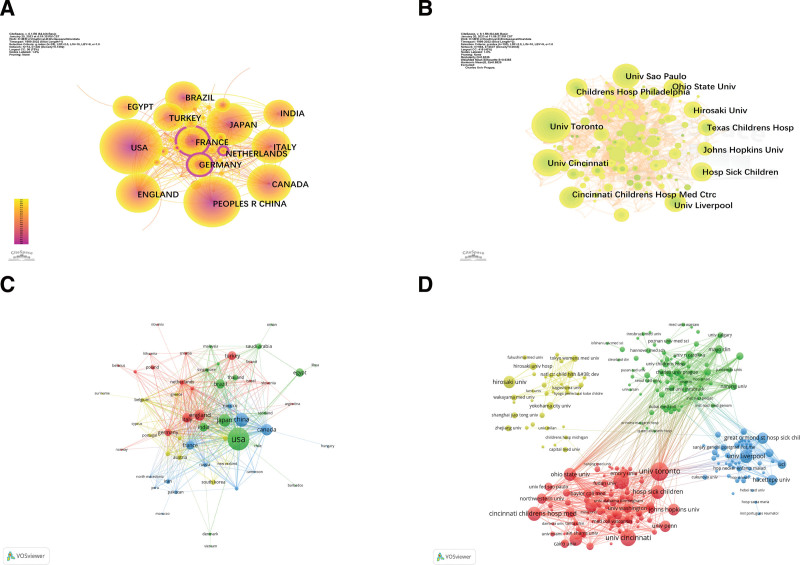
Analysis of the countries and institutions. (A) Network map of countries produced by the CiteSpace tool. (B) Network map of institutions produced by the CiteSpace tool. (C) Visualized the cooperation between countries in LN of children. (D) Visualized the cooperation between institutions in LN of children. Each node represents a country, the size of the node indicates the publication output of the country, and high centrality (>0.1) is represented by nodes with purple rings.

### 3.3. Analyze of journals and co-cited journals

**Figure 4.**
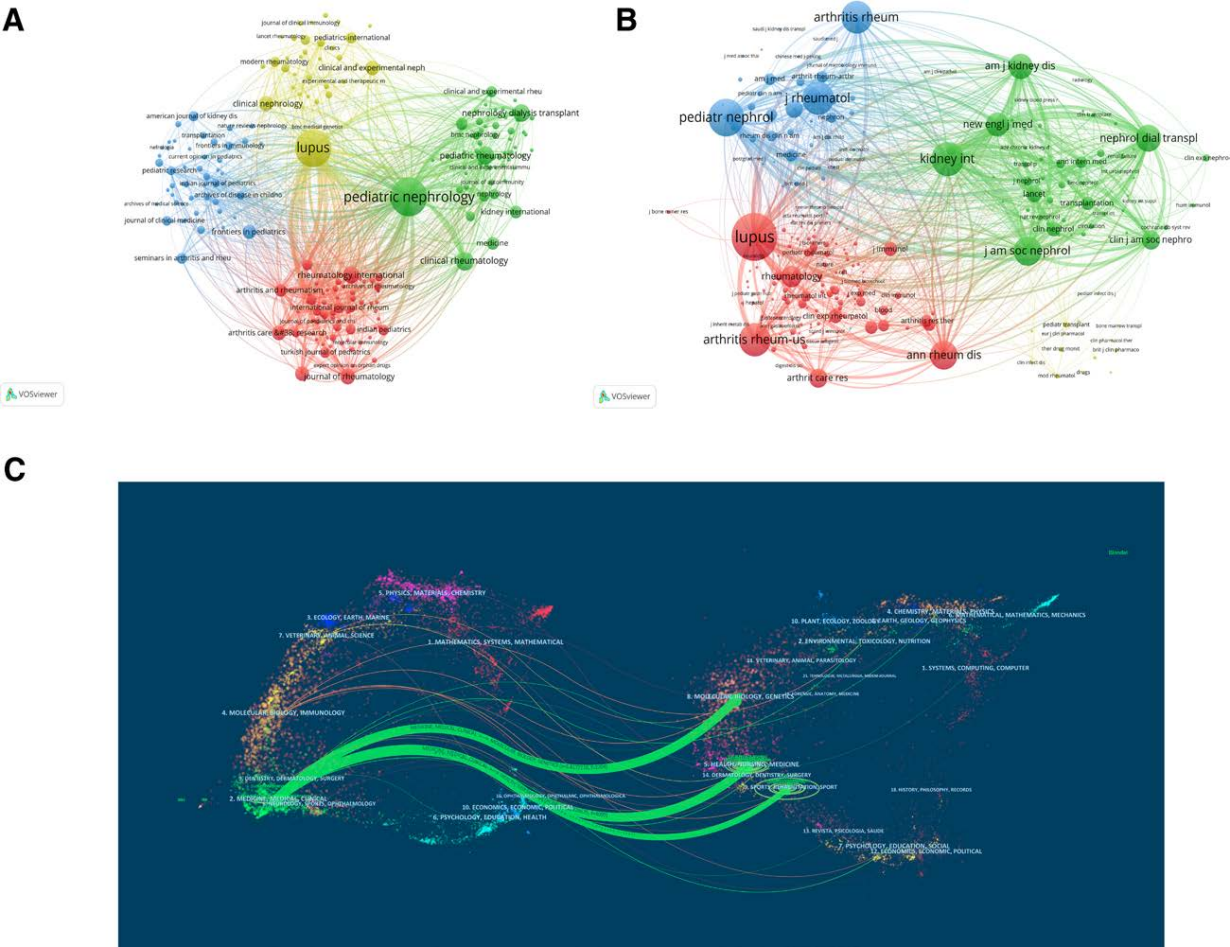
Analysis of the journals. (A) Network map of journals produced by the VOSviewer tool. (B) Network map of cited journals produced by the VOSviewer tool. The size of the node indicates the co-occurrence frequencies of journals, the link reflects the co-occurrence relationship between journals, and the same color of node represents the respective cluster class. (C) The dual-map overlay of LN in children research.

As shown in Tables 4 and 5, the most prolific journal was pediatric nephrology (IF 2.67) with 129 publications, followed by lupus (IF 2.21) with 126. Pediatric nephrology was classified as Q2, it indicates that this journal has a high academic reputation. In addition, lupus was cited the most frequently, followed by pediatr nephrology and Kidney International (IF 17.72), and the first 2 journals cited were also the most prolific. These results indicate that these 2 journals are more influential and authoritative than other journals in the field of LN in children. Figure 4A and B represent literatures and cited literatures, respectively. It can be seen from the figure that pediatric nephrology and lupus have the largest nodes, which also indicates that these 2 journals have the highest output and cited frequency. Figure 4C indicates the relationship between the journal and the cited journal. Green line represents from the Medicine/Medical/Clinical journals cited literature mostly from the Health/Nursing/Medicine/Rehabilitation/Sports/Molecular Biology/Genetics.

**Table 4:** Top 10 journals distributed by publications.

Rank	Journal	Publications	IF (JCR 2022)	JCR quartile
1	Pediatric Nephrology	129	2.67	Q2
2	Lupus	126	2.21	Q3
3	Clinical Rheumatology	28	3.10	Q3
4	Pediatric Rheumatology	26	2.27	Q3
5	Nephrology Dialysis Transplantation	25	5.51	Q1
6	Rheumatology International	23	3.67	Q2
7	Journal of Rheumatology	19	3.31	Q2
8	Clinical Nephrology	18	0.95	Q4
9	Arthritis Care & Research	17	4.46	Q2
10	Rheumatology	17	4.70	Q1

**Table 5:** Top 10 journals distributed by citations.

Rank	Cited journal	Total citations	IF (JCR 2022)	JCR quartile
1	Lupus	2710	2.21	Q3
2	Pediatric Nephrology	1813	2.67	Q2
3	Kidney International	1555	17.72	Q1
4	Journal of Rheumatology	1489	3.31	Q2
5	Arthritis rheum-us	1480	–	–
6	Arthritis & Rheumatology	1451	12.00	Q1
7	Journal of the American Society of Nephrology	1238	11.9399	Q1
8	Annals of the Rheumatic Diseases	1186	23.9476	Q1
9	Nephrology Dialysis Transplantation	1079	5.5161	Q1
10	American Journal of Kidney Diseases	980	11.8165	Q1

### 3.4. Analyze of keywords

**Figure 5.**
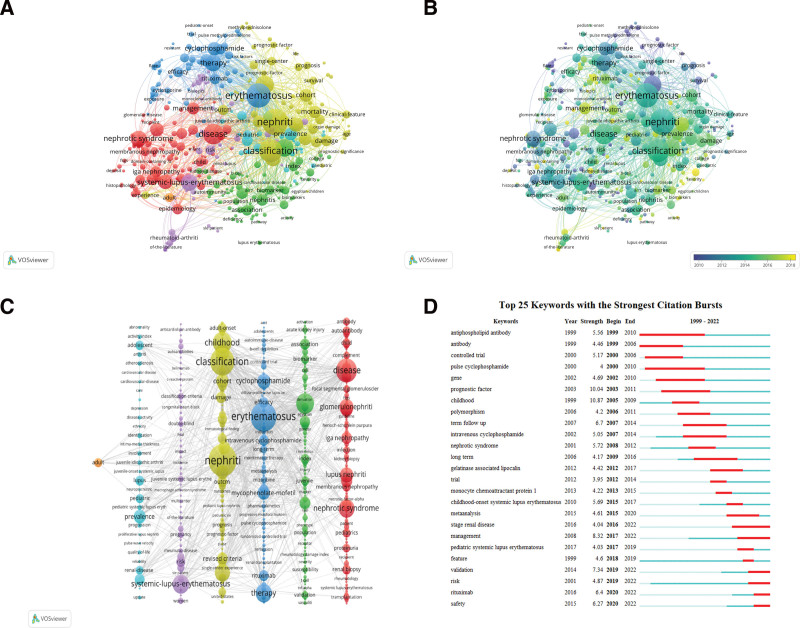
Analyze of keywords. (A) Visualization of keywords. (B). Timeline view of the keywords. (C) Clustering map of keywords. (D) The top 25 keywords with the strongest citation bursts.

Keyword co-occurrence network helps us to understand the research hotspot and research trend in this field. Table 6 shows the top 10 keywords that appeared frequently in the past 23 years, except the subject words Children and LN; in addition to systemic lupus erythematosus, nephriti appeared the most frequently, followed by erythematosus and classification. Visualization by VOSviewer except the subject words children and LN; On the distribution of systemic lupus erythematosus, it can be seen from Figure 5A that the node of nephriti is the largest, followed by erythematosus. Figure 5B is the time graph of keywords. The darker the color, the earlier the time, and the closer to yellow the later, representing the frontier hot spots in this field. Figure 5C can be divided into 7 clusters according to color. Red is disease/LN/nephrotic syndrome/renal biopsy. Green is a disease—activity/nephrity/expression/association; dark blue is erythematosus/therapy/cyclophosphamide/mycophenolate mofetil; yellow is nephriti/classification/childhood/SLE; purple is a systemic lupus erythematosus/risk/classification criteria; light blue is prevalence/lupus/pediatric/adolescent; orange is adult. We analyzed the top 25 keywords in burst intensity, mainly focusing on 3 aspects: laboratory examination, drug therapy and long-term prognosis. As shown in Figure 5D,the results showed that, antiphospholipid antibody first appears with burst intensity, childhood with the highest burst intensity, and antiphospholipid antibody with the longest burst duration. This suggests that the word is a hot research topic in the field of child with LN, look down, keywords from the start to appear and continue to burst to now have the validation/risk/rituximab/safety, this suggests that the key word is current research hotspot.

**Table 6:** Top 10 keywords with the highest frequency of occurrence.

Rank	Keywords	Occurrences
1	Children	402
2	Systemic lupus erythematosus	304
3	Lupus nephritis	243
4	Nephriti	232
5	Erythematosus	203
6	Classification	185
7	Disease	150
8	Childhood	129
9	Disease-activity	118
10	Systemic-lupus-erythematosus	107

### 3.5. Analyze of co-cited references and references burst

**Figure 6.**
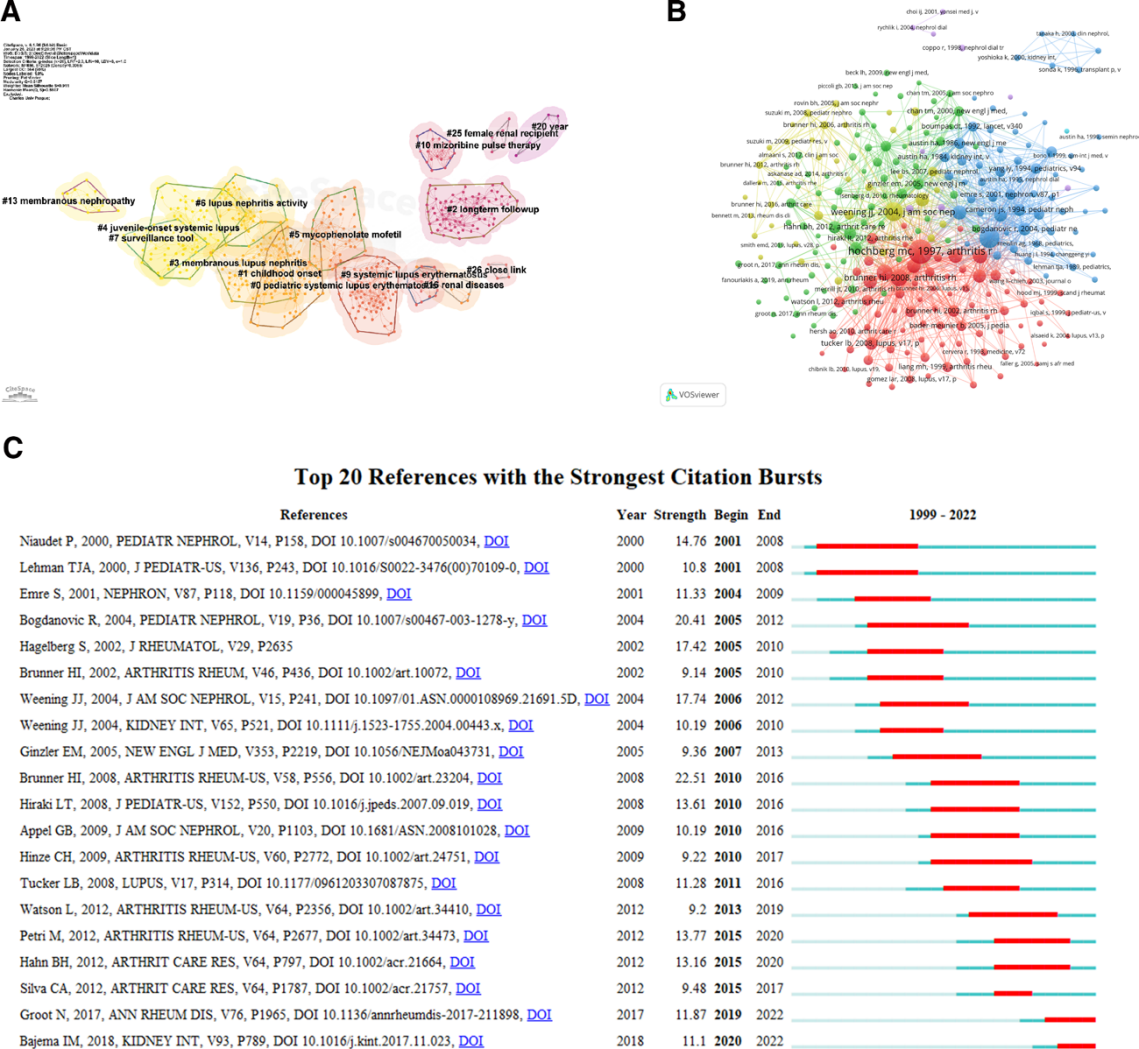
Analyze of co-cited references and references burst. (A) Clustering map of reference co-citation related to research of LN in children. (B). Visualization of co-cited references. (C) The top 20 references with the strongest citation bursts.

The co-citations of references were analyzed in combination with Table 7 and Figure 6. As shown in Table 6 and Figure 6B, the most cited references were Hochberg MCMCS article published in Arthritis Rheum in 1997, with 236 citations. This paper updated the classification criteria for systemic lupus erythematosus revised by the American College of Rheumatology in 1982, and proposed more rigorous criteria for biomarkers of lupus.^[21]^ The second most frequently cited article was published in 1982 Arthritis Rheum, which was also a guideline article. It summarized the diagnostic criteria of systemic lupus erythematosus accurately and succinctly, and added new serological criteria such as antinuclear antibody detection, DNA antibody detection and Simith antibody detection. It provides a great reference value for the study of systemic lupus erythematosus and its complications for later generations.^[22]^ As can be seen from the table, the earlier the article was published, the more times it was cited. Figure 6C shows that the burst intensity of the literature published by Brunner HI in 2008 in ARTHRITIS RHEUM-US is the strongest, and the number of citations of this paper also ranks the fourth. Through prospective studies, this paper confirmed that compared with adult systemic lupus erythematosus patients with childhood systemic lupus erythematosus, The incidence of kidney disease is higher, and there is a tendency of proliferative nephritis in children with systemic lupus erythematosus.^[3]^ In 2013, the authors made a supplement to the differences between children and adults with systemic lupus erythematosus. The risk of anti-DSDNA antibody positive in children with systemic lupus erythematosus was higher, and anticardiolipin positive was significantly higher in patients with childhood systemic lupus erythematosus than in patients with aSLE. Children with lupus have a higher risk of developing LN than adults with lupus.^[23]^ Citespace provides cluster analysis of co-citation literature, which enables us to more intuitively see the trend of co-citation of literature and hot research topics. Therefore, Citespace is applied to cluster analysis of literature, and the ModularityQ is 0.8157. Weighted mean Silhouette is 26 clustering of 0.911, and literatures in the same cluster are homogenous, as shown in Figure 6A. On the one hand, pediatric systemic lupus erythematosus is included in the disease type. #3 membranous LN, #6 LN activity, #7 surveillance tool, #9 systemic lupus erythematosus, #13 nephropathy, #16 renal diseases. The second aspect is disease onset and prognosis, including #1 childhood onset, #4 juvenile-onset systemic lupus, #20 year, #25 female renal recipient, the third aspect is the treatment and monitoring of diseases including #2 long-term follow-up, #5 mycophenolate mofetil, #10 mizoribine pulse therapy, #26 close link.

**Table 7:** Top 10 cited reference with the highest citations of occurrence.

Rank	Cited reference	Citations
1	Hochberg MC, 1997, Arthritis Rheumatol, v40, p1725	236
2	Tan EM, 1982, Arthritis Rheumatol, v25, p1271	164
3	Weening JJ. 2004, J Am Soc Nephrol,v15, p241	139
4	Brunner HI, 2008, Arthritis Rheum-US, v58, p556	121
5	Gladman DD, 2002,J Rheumatol, v29, p288	107
6	Tucker LB, 1995, Brit J Rheumatol, v34, p866	89
7	Hagelberg S, 2002, J Rheumatol, v29, p2635	83
8	Bombardier C, 1992, Arthritis Rheumatol, v35, p630	81
9	Hiraki LT, 2008, J Pediatr-US, v152, p550	78
10	Petri M, 2012, Arthritis Rheum-US, v64, p2677	76

## 4. Discussion

In this study, CtieSpace and VOSviewer were used for the first time to visually analyze the literature in the field of LN in children, and the research frontiers and hot spots in this field were summarized. This study collected a total of 1059 literatures from January 1, 1999 to November 17, 2022. The publication trend of literatures is increasing year by year, and the research enthusiasm of researchers in this field is only increasing.

The United States dominates in the number of papers published, the number of authors, the field of institutions and the cooperation between countries. Among the top 10 authors in the number of published papers, 5 are from the United States, and among the top 10 institutions, 5 are located in the United States. It is enough to show that the influence of the United States in the field of LN in children is huge and occupies the forefront of the research in this field. Among the top 10 countries in terms of literature quantity, only China and India are developing countries, while the rest are developed countries. In future research, cooperation between countries should be enhanced. In the analysis of authors, Brunner and Hermine I ranked first in terms of the number of articles published and the number of cited articles, which fully indicated that he had high authority and influence in the field of LN in children. In the analysis of journals, we found that most published journals belong to Q2/Q3 partition, and most cited journals come from Q1 partition, indicating that literatures in Q1 partition have greater authority and influence in this field. The results of this study reveal researchers, journals, research institutions and countries with higher influence, providing guidance for future cooperation among researchers.

Comprehensive analysis of keywords and references. We have found that antiphospholipid antibody/pulse cyclophosphamide/gene from 1999 is associated with biomarkers, genes, treatment has gradually changed to management/risk/safety related to the prognosis of the disease in recent years. There are many biomarkers for LN, such as antiphospholipid antibody, urinary neutrophil gelatinase-associated lipid carrier protein, urinary CD163, etc, but most of these biomarkers are related to diagnosis or therapeutic response. So far, no biomarker can predict the occurrence of LN in preSLE. Antiphospholipid antibodies (APLa) are a heterogeneous family of antibodies, including anticardiolipin antibodies, lupus anticoagulant, and anti-β2-glycoprotein I, which are positive in 30% to 40% of patients with systemic lupus erythematosus.^[24]^ APLa positive SLE patients are at higher risk for LN.^[25]^ Sulaiman M conducted APLa detection on 59 patients with LN diagnosed by kidney biopsy, and found that about 78% of children with LN were APLa positive, and APLa positive patients had a higher probability of developing renal microthrombin than APLa negative patients. The pathological type of nephritis in patients with intrarenal microthrombus is proliferative glomerulonephritis.^[26]^ This is consistent with the conclusion that Xiaokai Ding APLa plays a greater role in proliferative glomerulonephritis than in membranous proliferative glomerulonephritis.^[24]^ Gabriella Moroni^[27]^ and DYH Yap^[28]^ found that the kidney survival rate of LN patients with antiphospholipid antibody positive was lower than that of patients with antiphospholipid and anti-negative form. In addition, there were still many literatures in this field^[29,30]^ that studied the role of antiphospholipid antibody in LN. However, so far, no studies have confirmed that antiphospholipid antibodies can predict the occurrence and prognosis of LN, which may become a hot topic in future research. Another biomarker, monocyte chemotactic protein-1 (MCP-1), a member of the chemokine superfamily synthesized and secreted by monocytes/macrophages, kidney cells and fibroblasts, is widely expressed and closely related to the occurrence and development of kidney disease.^[31]^ The expression of MCP-1 can not only recruit and activate more inflammatory cells to penetrate the kidney. It can also directly stimulate the immune cells of the kidney, promote the secretion of inflammatory mediators, promote the synthesis of extracellular matrix, and aggravate the pathological injury process of LN.^[32]^ Studies have confirmed that MCP-1 promotes renal tubulointerstitial damage, but does not promote glomerular damage.^[33]^ Studies have confirmed that serum and urinary MCP-1 levels in patients with LN are significantly elevated and moderately significantly positively correlated with 24-hour urinary protein, anti-DSDNA antibody, renal SLEDAI, and biopsy activity index, while uMCP-1 is moderately significantly negatively correlated with serum albumin.^[32]^ In recent years, the focus of pediatric LN has gradually shifted to the management, prognosis and safety of the disease, which also indicates that the field of pediatric LN in the diagnosis and treatment of the disease itself may reach a bottleneck. We expect that this study will lead to further research in the field of LN in children.

## 5. Advantages and limitations

No researchers have published a bibliometric analysis of LN studies. In this study, Citespace (6.1.R6) and VOSviewer (1.6.18) were used to conduct data statistics and frontier hot spot analysis on LN from the perspective of bibliometrics, providing a meaningful reference for the research in this field. The study is incomplete and has its limitations. First, we did not collect literature from other databases such as Pubmed, Embase, and Scopus. Second, we only kept the English literature. Finally, VOSviewer and CiteSpace are not able to perform higher level statistical analysis, which can cause statistical bias. However, these restrictions do not affect the comprehensive information extraction and analysis of LN.

## 6. Conclusions

This study provides knowledge about childhood LN from the perspective of bibliometrics. The results show that researchers are increasingly interested in the study of childhood LN, and there is still a high enthusiasm for the study of biomarkers related to the diagnosis and treatment of childhood LN. However, there are still some unsolved problems in this field. Future research focus should focus on how to effectively detect and prevent LN under noninvasive operation. However, the quality of life of children with lupus cannot be ignored. This will also become a research hotspot in the future. However, there are still limitations in this study. First of all, only a single WOS database is selected, and the language is limited to English and the literature type is limited to article, which may lead to incomplete collection of articles. In addition, the software cannot run when there is too much data, which we believe can be solved with the efforts of the software research team.

## Author contributions

**Conceptualization:** Fei Luo, Yuan Zhang.

**Validation:** Juanjuan Diao.

**Writing – original draft:** Yunhong Ma, Shuangyi Wang.
